# High-entropy alloy electrocatalysts go to (sub-)nanoscale

**DOI:** 10.1126/sciadv.adn2877

**Published:** 2024-06-05

**Authors:** Menggang Li, Fangxu Lin, Shipeng Zhang, Rui Zhao, Lu Tao, Lu Li, Junyi Li, Lingyou Zeng, Mingchuan Luo, Shaojun Guo

**Affiliations:** ^1^ School of Materials Science and Engineering, Peking University, Beijing 100871, China.; ^2^ Beijing Innovation Centre for Engineering Science and Advanced Technology, Peking University, Beijing 100871, China.

## Abstract

Alloying has proven power to upgrade metallic electrocatalysts, while the traditional alloys encounter limitation for optimizing electronic structures of surface metallic sites in a continuous manner. High-entropy alloys (HEAs) overcome this limitation by manageably tuning the adsorption/desorption energies of reaction intermediates. Recently, the marriage of nanotechnology and HEAs has made considerable progresses for renewable energy technologies, showing two important trends of size diminishment and multidimensionality. This review is dedicated to summarizing recent advances of HEAs that are rationally designed for energy electrocatalysis. We first explain the advantages of HEAs as electrocatalysts from three aspects: high entropy, nanometer, and multidimension. Then, several structural regulation methods are proposed to promote the electrocatalysis of HEAs, involving the thermodynamically nonequilibrium synthesis, regulating the (sub-)nanosize and anisotropic morphologies, as well as engineering the atomic ordering. The general relationship between the electronic structures and electrocatalytic properties of HEAs is further discussed. Finally, we outline remaining challenges of this field, aiming to inspire more sophisticated HEA-based nanocatalysts.

## Teaser

Recent advances of high-entropy alloys that are rationally designed for energy electrocatalysis are systematically summarized.

## INTRODUCTION

The increasing energy consumption and environmental issues have presented a severe challenge to the sustainable development of human society (*1*). This stimulates the exploration of economical and eco-friendly energy generation, storage, and conversion involving the electrochemical processes (*2*). The suitable catalysts are necessary in these processes to lower the energy barrier and accelerate reaction kinetics to improve efficiency (*3*). Metal-based materials have occupied half of the catalysts in heterogeneous catalysis. For instance, platinum (Pt)–based materials show the most promising application in oxygen reduction reaction (ORR) and hydrogen evolution reaction (HER) electrocatalysis (*4*–*6*); ruthenium (Ru)–/iridium (Ir)–based materials are considered as the most effective electrocatalysts toward oxygen evolution reaction (OER) (*7*, *8*); copper (Cu)–based materials have been confirmed to be capable of producing C_2_/C_2+_ hydrocarbons with high selectivity in CO_2_ reduction reaction (CO_2_RR) electrocatalysis (*9*). However, a single metal site is not enough to show satisfactory activity and stability, necessitating the optimization of these metal catalysts for advanced electrocatalysis.

It is widely accepted that the adsorption/desorption behavior toward intermediates is the key to determining the electrocatalytic performance, which is closely related to the electronic and geometric structures of the catalysts (*10*). Alloying has provided a promising strategy to regulate the electronic structures of surface or near-surface atoms via a unique strain/ligand effect, thus creating a variety of bimetallic or trimetallic electrocatalysts (*11*–*13*). With the rapid development of nanotechnology, electrocatalytic activities of these catalysts have been substantially boosted by engineering geometric and electronic structures, including doping engineering (*14*) and interface engineering (*15*). However, the compositional range of the alloyed atoms is limited by the differences of crystal structure, atomic size, electronegativity, and electron concentration between alloyed atoms in metal catalysts (*16*–*18*). This leads to a limited regulation of the electronic structure and optimization of the adsorption energy toward intermediates, making it difficult to maximize the catalytic activity. To further break the scaling relationships for electrocatalysis, it is necessary to realize the continuous adjustment of the surface electronic structure of metal catalysts.

Since high-entropy alloys (HEAs) were developed as an important kind of alloy system in 2004 (*19*), the research upsurge of HEAs in the scientific and industrial community has been set off, including cryogenic applications, biomedical materials, aerospace engineering, and photothermal conversion (*20*–*23*). Continuous adjustment of component distribution makes it possible to break the barrier of traditional binary or ternary alloys, providing adsorption/desorption energy toward reaction intermediates closer to the optimal value (*24*–*26*). Therefore, HEAs are expected to show great potential in heterogeneous catalysis. Traditionally, HEAs prepared by top-down approach, such as mechanical alloying, high-energy ball milling, laser ablation, and arc melting, are all bulk, greatly losing the surface-active sites (*27*–*29*). This is not suitable for heterogeneous catalytic reactions. Recently, nanotechnologies have stimulated the continuously decreased size of catalysts, leading to many exposed atoms on the material surface to increase the possibility of catalytic reaction occurrence. The concept of nanomaterials can be introduced into HEAs to construct high-entropy nanoalloys (HENAs), which has attracted extensive attention in electrocatalysis.

**Fig. 1.**
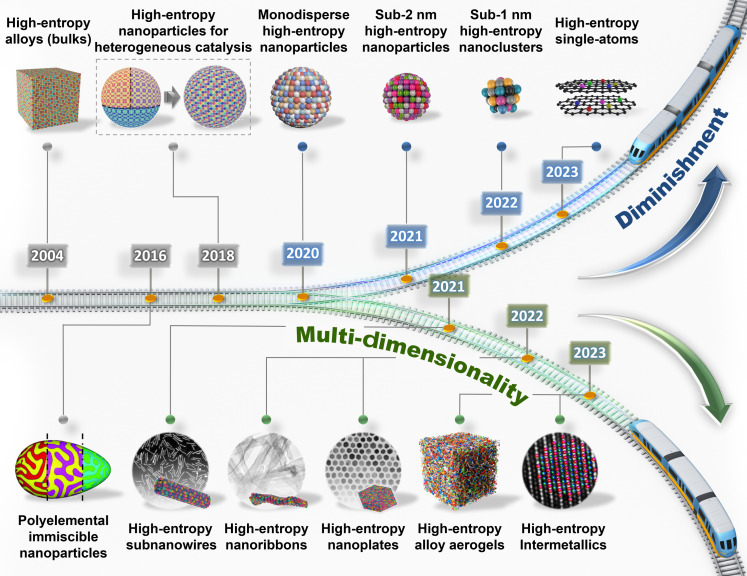
Historical timeline of breakthroughs in HEAs for energy electrocatalysis. Bulk HEAs were first presented in 2004. The data are reproduced with permission from John Wiley and Sons (*19*). A multielemental immiscible nanoparticle library with phase segregation was built in 2016. The data are reproduced with permission from the American Association for the Advancement of Science (*30*). Then, single-phase HEA nanoparticles were successfully synthesized for heterogeneous catalysis in 2018. The data are reproduced with permission from the American Association for the Advancement of Science (*31*). Afterward, HEA electrocatalysts began to develop along two important trends of diminishment and multidimensionality. Recently, subnanosized HEA nanoparticles and high-entropy single atoms have been developed as high-efficient electrocatalysts for HER, MOR, and ORR electrocatalysis. The data are reproduced with permission from the Nature Publishing Group (*32*), the American Chemical Society (*33*), the American Chemical Society (*34*), and the Nature Publishing Group (*35*). In addition, HEAs were designed from 0D to 3D, including subnanowires, nanoplates, nanoribbons, aerogels, and ordered intermetallics, and can serve as high-efficient electrocatalysts for HOR, MOR, and CO_2_RR electrocatalysis. The data are reproduced with permission from the Nature Publishing Group (*36*), John Wiley and Sons (*37*), the American Chemical Society (*38*), John Wiley and Sons (*39*), and the American Chemical Society (*40*).

Early reports have indicated that the polymetallic alloys tended to be phase-separated due to the intrinsic immiscibility of elements (*30*). However, driven by ultrafast thermodynamic nonequilibrium synthesis, single-phase HEA nanoparticles were successfully synthesized for heterogeneous catalysis, even for the immiscible elemental combinations (*31*). Since then, HEAs for energy electrocatalysis have shown two important trends of diminishment and multidimensionality (Fig. 1). In terms of diminishment, after HEA nanoparticles with a particle size of ~3.4 nm were made through a wet-chemical method (*32*), decreasing the size of HEA nanoparticles to 1.32 nm was well achieved by chemical co-reduction and continuous-flow liquid-phase reduction methods, respectively (*33*, *34*). Efficient HER electrocatalysis could be achieved by these HEA nanoparticles with gradually decreasing sizes. Very recently, five kinds of metal atoms were incorporated into nitrogen-doped carbon to achieve the atomization of high-entropy concepts (*35*). In addition, as the development of precise control of the HEA surface, a series of HEAs with anisotropic morphology have been developed, such as nanowires, nanoplates, nanoribbons, and aerogels, showing great potential in energy electrocatalysis (*36*–*39*). Note that the atomic arrangement of HEAs can be further regulated to present the concept of high-entropy intermetallic electrocatalysts, accelerating the electrocatalytic applications (*40*). Therefore, the developments of HEAs are beneficial for appropriately broadening the selectivity of the electrocatalysts.

This review aims to highlight recent advances in developing the HEAs for energy electrocatalysis over the past few years. The trends for the alloys for electrocatalysis have developed toward high entropy, nanometer, and multidimension. Starting from the advantages of HEAs in electrocatalysis, the rational structural designs of HEAs are summarized from the aspect of thermodynamically nonequilibrium synthesis, the regulation of the (sub-)nanosize and anisotropic morphologies, as well as the engineering of the atomic ordering and disordering. Specifically, diminishment and multidimensionality have been regarded as the two most important trends in developing HEA electrocatalysts. Using the typical electrocatalytic reactions as probes, such as water electrolyzer and fuel cell electrocatalysis, the application potential of HEAs in electrocatalysis is further demonstrated. The general relationship between the structures of HEAs and catalytic activity is discussed based on experimental observations and theoretical models. Finally, several major trends and challenges for future advanced studies will be presented to point out the direction for relevant researchers.

## ADVANTAGES OF HEA ELECTROCATALYSTS

HEAs represent a single-phase solid solution containing five or more dominant elements (*24*). Because of too many components, the structure of HEAs is relatively complicated, quite different from traditional alloys. In HEAs, different atoms randomly occupy lattice positions, promoting the formation of simple phase solid solutions. Moreover, the local order change induces the bond length and structural particularity. Therefore, HEAs show four characteristics different from traditional alloys: high-entropy, lattice distortion, sluggish diffusion, and cocktail effects. These four effects are the dominant reason why the HEAs are promising catalysts for electrochemical energy conversions. In addition, the desired catalysts used for electrocatalysis should be nanoscale. After all, traditional bulk materials lose too many active surface atoms. Hence, we also focus on the nano-size effect of HENAs. Finally, the anisotropically multidimensional structures can enhance anti-aggregation and Ostwald ripening during electrocatalytic processes compared with 0D counterparts. The stronger structural stability and increasing surface-active sites make the HEAs develop along the multidimensional trend, with the aim of maximizing electrocatalytic activity. In this section, we will summarize the advantages of HEAs in electrocatalysis from three aspects: high entropy, nanometer, and multidimension.

### From multimetal to HEAs

Different from traditional multimetal alloys, HEAs are subject to the dual constraints of elemental compositions and entropy. Hence, two kinds of definitions for HEAs, based on compositional and entropic concepts, respectively, were presented. On the basis of composition, the first definition indicates that HEAs contain five or more dominant elements, of which the atomic percentage of constituent elements varies from 5% to 35% from a broad point of view (*19*). Occasionally, there are a few trace elements with atomic percentage of less than 5%. The formation of long-range ordered structures is energetically prohibited by high mixing entropy. As a result, HEAs always form in a single-phase solid solution structure.

The other definition is based on the entropy. The total mixing entropy of HEAs (Δ*S*_mix_) generally includes the configurational, vibrational, magnetic dipole, and electronic randomness entropy, among which the configurational entropy plays the dominant role. Particularly, Δ*S*_mix_ is a function of the number of alloy components. Hence, the entropy-defined HEAs can be identified by Δ*S*_mix_. Here, the Δ*S*_mix_ of 1.5*R* can be used as the boundary between HEAs and medium-entropy alloys (MEAs) and that of 1.0*R* for medium- and low-entropy alloys (LEAs) (*24*). Considering the total alloy systems, the alloys constituted by more elements may strengthen the entropy driving force (*41*). Increasing Δ*S*_mix_ of HEAs induces a decreased free energy of alloys, thus leading to higher stability. However, the definition based on entropy is still controversial, because many alloys with less than five elements still present a single-phase solid solution structure, such as CrCoNi and FeCrCoNi (*42*, *43*). All in all, alloys containing at least five elements with high atomic percentage (composition-based definition) or with Δ*S*_mix_ larger than 1.5*R* (entropy-based definition) can be regarded as HEAs. In this case, the multimetal alloys have been successfully developed into HEAs via entropy engineering.

**Fig. 2.**
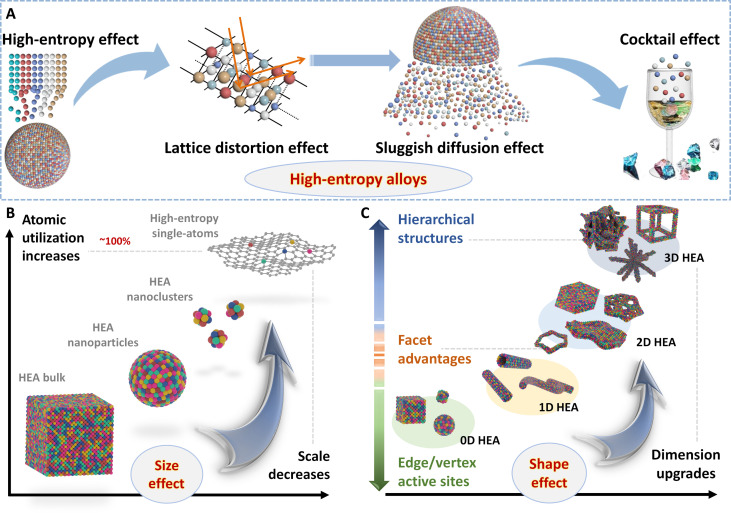
The advantages of HEAs for energy electrocatalysis. Schematic illustrations for (**A**) several effects of HEAs, (**B**) HEAs evolving from bulk to nanoscale, cluster, and atomic scale, and (**C**) HEAs evolving from nanoscale 0D to 1D, 2D, and 3D nanostructures.

The complicated components enable HEAs to generate many unexpected properties, including high-entropy, lattice distortion, sluggish diffusion, and cocktail effects (Fig. 2A). High-entropy effect refers to the high mixing entropy of alloys containing five or more elements uniformly, tending to form the single-phase solid solution instead of intermetallics. The high mixing entropy reduces the free energy of the solid-solution phase and promotes the formation of HEAs at higher temperatures. The number of phases in HEAs is reduced, and as a result, the stable solid-solution phase forming at higher temperatures is feasible (*19*). Therefore, the high-entropy effect explains why the phase number of HEAs is much smaller than that calculated by the simplified theoretical prediction. Especially, because the continuous regulation of compositions induced by the widened *d*-band electrons enhances the stability of HEAs, the high-entropy effect ensures that HEAs show unexpected catalytic performance.

The size difference between various atoms in HEAs inevitably leads to the deviation of the lattice matrix from the ideal positions, generating severe lattice distortion. In addition, the bond energy and crystal structure between different elements are also the main causes of lattice distortion. The lattice distortion effect not only optimizes the hardness, electrical, and thermal conductivity of HEAs but also dramatically improves their chemical properties (*44*–*46*). The mismatched atoms induce the thermodynamically nonequilibrium states of HEAs, guaranteeing the HEAs with higher potential energy and reducing the energy barrier for catalytic reactions.

The sluggish diffusion effect is a phenomenon that atoms in HEAs show a slower diffusion rate compared with those in traditional alloys. This is mainly because severe lattice distortion increases the energy barrier for atoms to be activated and migrated, thus reducing the efficient diffusion rate of atoms. This effect can be understood from two perspectives. First, atoms tend to jump into vacancies, widely existing in thermal equilibrium crystals above absolute zero, to achieve atom migration during diffusion. However, the difference of atoms before and after jumping into vacancies results in diverse bonding and local energies, and the atoms will be captured when jumping into a low-energy site. On the contrary, the high-energy site leads to the failed atomic migration. Nevertheless, the sluggish diffusion effect is inconspicuous in traditional alloys, because the local atomic configurations before and after atoms enter vacancies are similar (*47*). Additionally, the difference in diffusion rate for each element in HEAs is another reason for the sluggish diffusion effect. Diffusion behavior in alloys is the result of the coordinated movement of a large number of different atoms. Some elements are not as active as others, so jumping into the vacancies is difficult. Therefore, the slow diffusion of elements in HEAs becomes a rate-limiting factor in limiting the overall diffusion rate of the alloys. The sluggish diffusion effect endows HEAs with better phase stability, high-temperature strength, and creep resistance, thus providing excellent durability and strong structural stability during electrocatalytic processes.

The cocktail effect in HEAs refers to the unexpected synergies originating from the interaction between mixing multiple components. Specifically, the comprehensive performance of HEAs comes not only from the basic properties of single elements but also from the interaction between different elements. Therefore, mixing multielements may create special properties that a single element does not have. The cocktail effect means a synergistic mixing to produce an unpredictable result, and may even be greater than the sum of each principal component. Originally, the cocktail effect was used to explain the unexpected physicochemical properties (*48*). Recently, the enhanced catalytic performance has been well explained by the synergistic mechanism of multicomponent mixing in HEAs (*49*–*51*).

### From bulk to (sub)-nanometer

It is divided into four scales around the material world, including micrometer, submicrometer, nanometer, and atomic cluster. As for the traditional bulk materials, the finite number of surface atoms limits their applications in electrocatalysis. However, reducing the size of electrocatalytic materials to the nanoscale or even atomic scale can maximize the utilization of surface-active atoms, thus providing endless possibilities for developing electrocatalysis-related energy conversion devices. Note that the surface atomic exposure increases exponentially as the particle size of nanomaterials decreases (Fig. 2B). This stimulates the development of HEAs toward ultra-minimization. The small size, large surface/volume ratio, different surface chemical and electronic states from the inside of nanoparticles, and unsaturated surface atom coordination lead to more surface-active sites of nanoscale HEAs, making it suitable for electrocatalysis.

In addition, the nanoscale HEAs also exhibit unique properties similar to traditional nanomaterials, including discontinuity of electron energy levels, surface and interface effects, and quantum size and macroscopic quantum tunneling effects (*52*). These properties can be unified into nano-size effects, which play an important role in enhancing electrocatalysis by combining the advantages of HEAs. The nano-size effect refers to the phenomenon that the catalytic performance decreases/increases with the size increase of nanoparticles in electrocatalytic reactions. In terms of catalytic activity, nanoparticles should be as small as possible due to the larger proportion of surface-active atoms. However, strong surface energy will be induced by the too small size. To balance the high surface energy, adjacent nanoparticles will approach each other and agglomerate, resulting in the decline of their overall activity (*53*). Especially when the size of nanoparticles is below 2 nm, agglomeration is more likely to occur, even to a serious extent. This is also why commercial catalysts choose Pt nanoparticles with ~3 nm supported on carbon black. Extending to HEAs, researchers have been committed to developing HEA electrocatalysts with ultrasmall size, which not only maximizes the catalytic activity but also chooses appropriate methods to enhance stability when the size is too small (*32*–*35*, *54*–*56*).

### From zero to three-dimensions

As is well known, the 0D nanostructure is the most stable nanomaterials in thermodynamics, mainly because the truncated octahedron is the equilibrium shape for a single crystal with face-centered cubic (fcc) phases (*11*). Therefore, 0D nanoparticles are the easiest to synthesize and stabilize, especially under very strict preparation conditions. To obtain single-phase solid-solution HEAs, it is necessary to control the simultaneous reduction of metal atoms (*57*, *58*). This is highly dependent on high reduction temperature to promote the rapid reduction of all metal atoms and subsequent rapid cooling to maintain the thermodynamic nonequilibrium. Most HEAs are prepared based on this principle, while the rapid heating and cooling make the reaction conditions quite severe. Therefore, the current HEAs mainly exist in the form of stable nanoparticles. However, due to the limited surface-active sites and untailored surface electronic structures, 0D HEAs tend to exhibit low catalytic activity. Therefore, the HEA nanocatalysts with tailored anisotropic morphologies are the future pursuit for boosting electrocatalysis.

Compared with 0D nanomaterials, anisotropic nanostructures can provide multiple anchoring points toward supports, thus exhibiting many unique properties to realize high electrocatalytic performance, such as enhanced electron and mass transport, as well as improved chemical stability (*59*). Hence, nanocatalysts with anisotropic morphologies may show attractive catalytic applications. In this case, developing HEAs into these specific structures can generate more effects in heterogeneous catalysis because of more exposed low-coordinated atoms at the perimeters, as well as higher surface area. However, metals tend to form Wulff polyhedrons to minimize the surface energy of a specific facet; thereby, the intrinsically nonlayered metals and binary/ternary alloys have difficulty of spontaneously forming these anisotropic structures, let alone HEAs containing so many metal elements (*60*). However, with the rapid development of nanotechnology, a large number of HEAs with various morphologies have been successfully designed and developed to manipulate substantially enhanced electrocatalysis (Fig. 2C) (*36*–*39*, *61*–*64*).

## ADVANCED CHARACTERIZATIONS FOR HEAS

HEAs have shown potential applications in energy electrocatalysis but require detailed structural analysis to understand the structure-performance relationship. The accurate analysis of the geometric and electronic configurations provides a guarantee for studying the mechanism of their enhanced electrocatalysis. To this end, HEAs have been finely studied to reveal their single-phase crystal structures, uniform chemical compositions, anisotropic morphologies, and unique chemical states. Taking the short history of HEAs into account, however, it remains a great challenge to precisely identify the geometric and electronic configurations of HEAs.

### Crystallographic characterizations

**Fig. 3.**
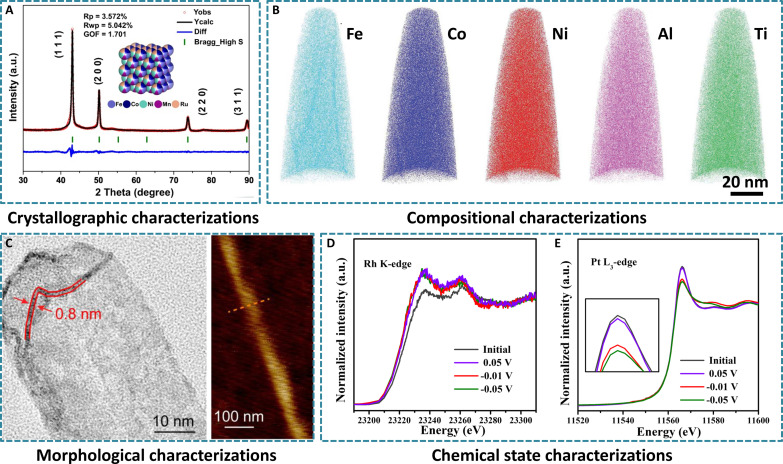
Characterizations of HEAs for energy electrocatalysis. (**A**) PXRD pattern of FeCoNiMnRu HEA nanoparticles with detailed Rietveld refinements. Reproduced with permission from the Nature Publishing Group (*65*). (**B**) 3D-APT images of FeCoNiAlTi HEIs. Reproduced with permission from John Wiley and Sons (*71*). (**C**) Representative HRTEM image and AFM image of PtPdIrRuAg HEA subnanometer ribbons. Reproduced with permission from the American Chemical Society (*38*). In situ normalized XANES spectra of RhPtFeCoNi HEA nanoparticles recorded at different potentials in fluorescence mode at the (**D**) Rh K-edge and (**E**) Pt L_3_-edge. Reproduced with permission from the American Chemical Society (*33*).

Powder x-ray diffraction (PXRD) patterns are the most powerful method to determine materials’ phase structures, including HEAs through peak positions and intensities. Most HEAs show a single-phase structure, while a minority of them exhibit biphasic structures, showing additional diffraction patterns (*65*–*67*). The ideal PXRD pattern of HEAs should display the diffraction peak of single-phase solid solution, rather than that of phase separation. For example, a typical PXRD pattern of FeCoNiMnRu HEAs reveals their single-phase fcc structure (Fig. 3A) (*65*). In addition, PXRD patterns can also be used to evaluate the particle size, lattice parameter, and degree of crystallinity by fitting the profile of the diffraction data (*68*). Moreover, the formation of the alloy phase can also be proved by the Fourier-transformed extended x-ray absorption fine structure (FT-EXAFS), mainly reflected by the shifted positions of metal-metal bonds in HEAs relative to the corresponding pure metals (*69*). In addition, atomic-level high-resolution transmission electron microscopy (HRTEM) and corresponding fast Fourier transform (FFT) patterns can further assist in identifying the crystal structure. For example, by identifying (110), (101), and (211) lattice planes from HRTEM image and FFT patterns, the body-centered cubic (bcc) phase structure of HEA nanoparticles containing 21 elements can be confirmed (*70*).

### Compositional characterizations

HEAs usually comprise more than four kinds of elements. Therefore, it is of notable to identify and quantify the elements, which can generally be accurately achieved by inductively coupled plasma–optical emission spectrometry (ICP-OES) and inductively coupled plasma–mass spectroscopy (ICP-MS). In addition, energy-dispersive x-ray spectroscopy (EDS), associated with electron microscopy, can also analyze the located elemental compositions in a selected area. However, they cannot give the specific distribution states of elements in HEAs. Elemental mappings, such as scanning electron microscopy–EDS (SEM-EDS) and scanning TEM–EDS (STEM-EDS), can offset this disadvantage to depict the detailed element distribution. The atomic-scale high-angle annular dark-field STEM-EDS (HAADF-STEM-EDS) can be used especially for the more accurate observation of the atomic distributions of elements (*70*). Linear scanning EDS profile is another tool to determine the atomic ratio of constituent elements by the peak intensity. Beyond these traditional compositional characterizations, the atomic chemical distributions of a high-entropy intermetallic (HEI) were further analyzed by three-dimensional atomic probe tomography (3D-APT) technique, where Fe, Co, Ni, Al, and Ti elements are uniformly distributed in HEIs (Fig. 3B) (*71*). X-ray photoelectron spectroscopy (XPS) can also evaluate the surface composition of materials, and for ultrasmall or ultrathin HEAs, it is also a powerful means to quantify the element compositions of HEAs accurately (*59*).

### Microstructural and morphological characterizations

With the rapid development of controlled synthesis of HEAs, it is critical to directly observe their microstructures and morphologies. SEM, TEM, and HAADF-STEM are all used for directly observing the microstructures of HEAs, showing the sizes, surface morphologies, and exposed crystal planes. For example, the HAADF-STEM image displays the nanowire morphology of PtRhMoRuIr HEAs with an average diameter of ~1 nm (*72*). For 2D structures, beyond the electron microscopies, atomic force microscopy (AFM) analysis can also reveal the thickness of 2D HEAs. As expected, the thickness of two overlapped PtPdIrRuAg HEA subnanometer ribbon layers was evaluated to be ~1.5 nm as revealed by the AFM image, in line with the single-layer result from high-magnification TEM image (Fig. 3C) (*38*). Additionally, Brunauer-Emmett-Teller (BET) and Barrett-Joyner-Halenda (BJH) measurements are very important to determine the specific surface area, pore volume, and pore size distribution of porous HEAs (*73*).

### Chemical state characterizations

Electrocatalysis often occurs on the surface of catalysts and at the interface with the electrolyte. The study on the surface chemical states contributed to deep understanding of the mechanisms of electrocatalytic reactions. XPS analysis has been considered as the most common characterizations to visualize the surface chemical states of HEAs, which is mainly achieved by detecting the kinetic energy of photoelectrons emitted from surface atoms. Although most atoms in HEAs are in metallic states, the surface elements are oxidized due to the long-term operations during electrocatalysis (*74*). More advanced, x-ray absorption spectroscopy (XAS), including x-ray absorption near-edge structure (XANES) and extended x-ray absorption spectroscopy fine structure (EXAFS), can provide more accurate chemical bond and atomic coordination information (*75*). For example, the change of electronic structures of RhPtFeCoNi HEA nanoparticles during HER electrocatalysis can be revealed by the operando XAS (*33*). On the basis of unchanged dominant features of XANES spectra for all applied voltages, Rh sites exhibited the negative shift toward lower energy compared with the other four elements, thus serving as the electron acceptor (Fig. 3D). At −0.01 V, the potential at which HER starts to react, the Pt intensity of XANES declined sharply, indicating that the main active sites in RhPtFeCoNi HEA nanoparticles are Rh and Pt (Fig. 3E). This in situ diagnostic strategy provides more vital structural or chemical information at the atomic level, so a more comprehensive understanding of the target material system’s structure/composition/valence information can be achieved. Therefore, it is anticipated that constructing a more feasible structure-performance relationship can guide more advanced design of HEAs in electrocatalytic future.

## STRUCTURAL REGULATION OF HEAS

In addition to the entropic effects, structural regulation also plays dominant role in the enhanced electrocatalysis of HEAs. As discussed above, most HEAs can be synthesized by the rapid reduction and cooling of metal atoms to maintain thermodynamic nonequilibrium, especially for the constituted elements with large differences in sizes and structures. However, these conventional thermodynamically nonequilibrium syntheses make it difficult to maintain the unique anisotropic structures of HEAs. Note that exerting certain kinds of reaction conditions onto the preparation process can drive the reduction potentials of different metals to equilibrium. This can not only control the uniformity of HEA nanocrystals well but also adjust the morphology and ordering degree of HEAs. In this section, we will discuss the structural design of HEAs based on the reported preparation strategies. The emphases for our discussion are the formation mechanism of HEAs in the nanoscale or atomic scale.

### Thermodynamically nonequilibrium synthesis

**Fig. 4.**
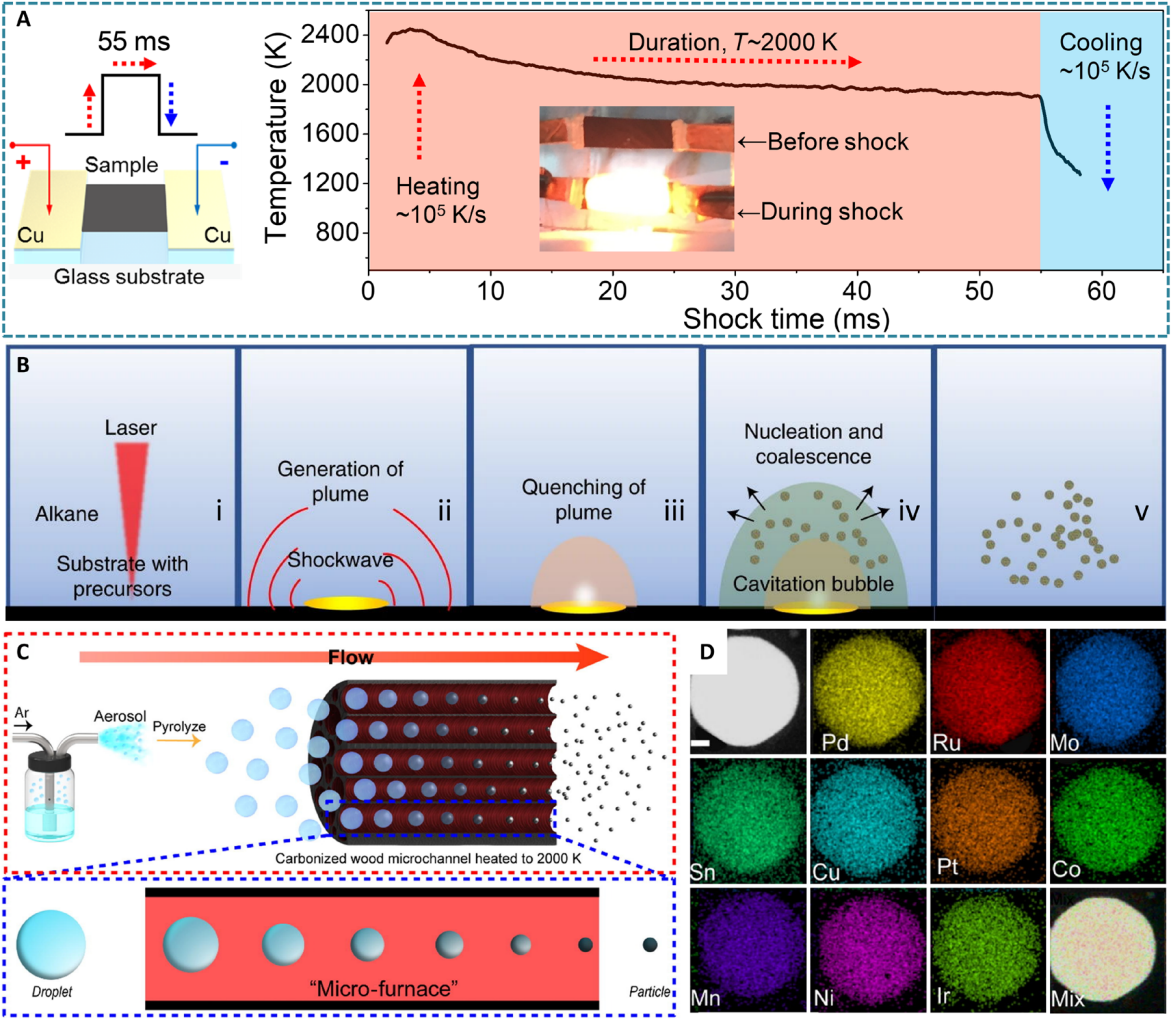
Synthesis of HEAs by nonequilibrium methods for efficient electrocatalysis. (**A**) Carbothermal shock strategy and the temporal evolution of temperature during the 55-ms thermal shock of HEA nanoparticles. Reproduced with permission from the American Association for the Advancement of Science (*31*). (**B**) Schematic illustration for the formation mechanism of HEA nanoparticles by the laser scanning ablation method [this synthesis involves the sequential processes of (i) pulse penetration, (ii) pulse absorption, (iii) plume expansion, (iv) bubble generation, and (v) bubble collapse]. Reproduced with permission from the Nature Publishing Group (*76*). (**C**) Schematic illustration for the droplets transporting through the carbonized wood microchannels heated to 2000 K via the carrier gas and the magnified wood microchannel. (**D**) EDS elemental mapping images of a 10-element AuCoCuIrPtMnMoNiPdRu HEA nanoparticle (scale bar, 20 nm). Reproduced with permission from Elsevier (*84*).

To promote the mixing of metals with different chemical properties, thermodynamically nonequilibrium environments are necessary, often realized by the extremely high-temperature and high-pressure environment. This synthesis is generally achieved through fast material formation process to prevent atom diffusion, thus leaving immiscible elements mixed in a metastable alloy phase. Therefore, this strategy has been considered as the most efficient method to prepare the HEAs. The common nonequilibrium syntheses include high-temperature shockwave, laser scanning ablation, and electrical explosion (*31*, *76*–*79*). A typical preparation method for HEA nanoparticles is carbothermal shock method that heats the metals to an extremely high temperature of 2000 K for 55 ms, followed by an exceptional cooling rate of several milliseconds (Fig. 4A) (*31*). The flash heating and thermal shock processes make the metal salts the liquid metal droplets, driving particle fission and fusion with a higher frequency to the uniform mixing of multiple elements. In addition, the carbothermal shock duration and the selected carbon supports are the key to alleviating the coalescence of nanoparticles, and the subsequent cooling in a twinkling ensures the elimination of solute partitioning during the formation of HEAs. As a result, a HEA nanoparticle library containing up to eight dissimilar elements (Pt, Pd, Ni, Co, Fe, Au, Cu, and Sn) was built. However, a nonnegligible issue is that the limitations of selected supports acting as the electric conductive and heating elements still exist.

The laser scanning ablation can break the limitation of support materials to obtain more HEAs, because a laser pulse can accurately confine energy in the desired microregions of the supports (*80*). Using this method, a series of HEA nanoparticles supported on carbon substrates were synthesized, and the substrates can be extended into other thermally sensitive substrates, such as Cu foam and glass (*76*). Taking carbon nanofiber as an example, metal precursors with constant stoichiometry loaded on the substrates were transferred into liquid alkane and irradiated by laser pulses at ~25°C. During this process, the laser pulse penetrated through the liquid phase induces the formation of plume at high temperature and high pressure. Then, the emission shockwave generated from the recoil of plume causes the plume to expand into the surrounding liquid and be rapidly cooled to nucleate nanoparticles (Fig. 4B). Because of the extremely fast synthesis with the pulse duration of only 5 ns, as many as nine metallic elements can be uniformly mixed, independent of their thermodynamic solubility.

Since light can interact with materials, the energy exchange between photons and electrons can also be generated by the photothermal effects. This will induce photo-excited electrons to produce thermic energy under the light-exposed supports, thus achieving fast and mass-scale synthesis of nanoparticles at thermodynamically nonequilibrium states. Recently, using the carbon nanofibers as the supports, HEA nanoparticles were synthesized by the millisecond-scale light-assisted flash-thermal shock, featuring the advantages of ambient air, large area, and remote (*78*). Typically, the surface temperature of carbon nanofibers can increase up to 1800°C within 5 ms due to the photon-electron couplings and energy exchange, providing enough energy to instantly decompose the precursors and splitting/fusion of liquid metal particles. Therefore, HEA nanoparticles with up to nine elements, including Pt, Ir, Fe, Ni, Co, La, Ce, In, and Sr, can be successfully synthesized, and the supports can be extended to any 2D nanomaterial with exposure properties, e.g., MXenes and graphene oxides.

In addition, by breaking through the thermodynamic immiscibility limit, other shock-type strategies were also developed, including the fast-moving bed pyrolysis, microwave heating method, and continuous “droplet-to-particle” method (*81*–*83*). However, the expensive and complex instruments, as well as the harsh preparation conditions of these strategies, make it impossible to spread widely. To simplify the preparation process, a droplet-to-particle aerosol technique combined with a high-temperature (~2000 K) microchannel reactor was adopted to overcome the limitations of traditional aerosol spray techniques (*84*). As shown in Fig. 4C, a collision nebulizer was applied to produce aerosolized droplets, in which metal precursors were uniformly mixed and brought into the microchannel of the carbonized wood reactor. After the reactor was powered on, the droplets were instantly heated to a high temperature to promote the formation of complex HEA nanoparticles containing 10 immiscible transition metal elements (Fig. 4D).

### Size regulation of HEAs

**Fig. 5.**
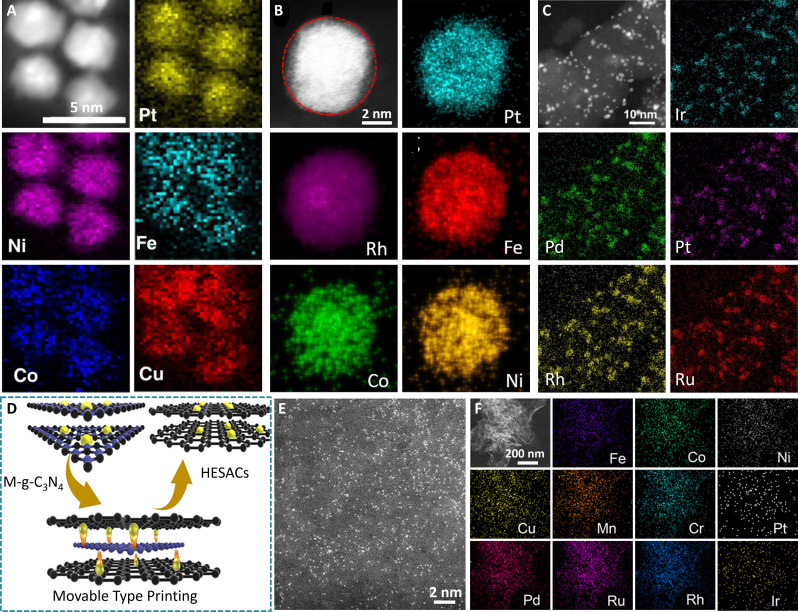
Size regulation of HEAs for efficient electrocatalysis. EDS elemental mapping images of (**A**) PtNiFeCoCu, (**B**) NiCoFePtRh, and (**C**) IrPdPtRhRu HEA nanoparticles. Reproduced with permission from the Nature Publishing Group (*32*), the American Chemical Society (*33*), and the American Chemical Society (*34*). (**D**) Schematic illustration for the formation of HESACs by a movable type printing method. (**E**) High-resolution HAADF-STEM image of quinary FeCoNiCuMn HESACs. (**F**) EDS elemental mapping images of undecimal FeCoNiCuMnCrPtPdRuRhIr HESACs. Reproduced with permission from the Nature Publishing Group (*54*).

Traditional thermodynamically nonequilibrium syntheses have difficulty of making metal atoms diffuse uniformly and slowly, leading to the nanoparticles with wide size distribution. The wet-chemical method, as a typical near-equilibrium approach, has been considered the most common approach to prepare alloys, which is realized by selecting a proper reductant and structure-directing agent. Therefore, it is easy to obtain monodisperse nanocrystals with controlled morphology. However, strongly immiscible elements are difficult to alloy, which can induce phase separation and heterogeneity (*67*, *85*). To address this issue, several transition metal elements (Ni, Fe, Co, and Cu) with similar atomic radius and lower heat of formation were selected to alloy with Pt to form stable HEA nanoparticles (*32*). In this synthesis, metal acetylacetonates are the key to the formation of solid solutions because that strong metal-acetylacetonate bond can slow down the precipitation rates of metals. As-prepared PtNiFeCoCu HEA nanoparticles are monodisperse with a diameter as small as 3.4 ± 0.6 nm (Fig. 5A), which are the advantages of wet-chemical methods. However, the metals with great differences in redox potentials are not mixed spontaneously with each other, such as Rh and Ag. By slowly adding low-concentration metal precursors into a heated reductant solution, immiscible metals can be reduced simultaneously (*86*). Since the initially reduced metal atoms have no choice but to aggregate to achieve stability, the difference in reduction rates can be negligible. According to this hypothesis, Pt group metal–based HEA nanoparticles (RuRhPdOsIrPt) containing immiscible metals were successfully obtained in a single-phase solid solution (*68*). These co-reduction strategies also have additional merit in that introducing surfactant and/or structure-directing agent can induce the growth of HEAs with special morphology or non-0D structures.

The metal atoms have different migration rates under high-temperature environments and thus will interdiffuse to facilitate the formation of single-phase alloys (*87*). The abovementioned sequential reduction strategy is also inspired by this mechanism, thereby eliminating the effect of segregation structures. Using intermetallic PdCu nanoparticles as seeds, PdCu/PtNiCo core/shell nanoparticles were first synthesized and then were annealed to induce the atomic mixing of metals to form PdCuPtNiCo HEA nanoparticles (*88*). Afterward, this strategy was further extended into other quinary (PdCuPtNiM, M = Ir, Rh, Fe, and Ru), senary (PdCuPtNiCoRh), and septenary (PdCuPtNiCoRhIr) HEA systems (*89*). Thermodynamically, the increased mixing entropy is beneficial to forming a single-phase solid solution; kinetically, the lattice distortion and constrained atomic diffusivity suppress the phase separation during the cooling process.

Reducing the size of 0D nanomaterials to subnanoscale, even to single-atom catalysts, can enhance the surface atomic utilization (*90*). In this case, HEA nanoparticles with sub–2 nm and sub–1 nm sizes have been successfully developed via the co-reduction of corresponding metal precursors (Fig. 5, B and C) (*33*, *34*). Furthermore, to maximize atomic utilizations, the HEA concept has been developed into multimetallic single-atom catalyst systems via a modified movable type printing method (*54*). Specifically, after the dopamine hydrochloride was coated on the printing templates anchored with metal precursors and melamine (M-g-C_3_N_4_), the metal atoms would be excited and escaped from M-g-C_3_N_4_ upon high pyrolysis temperature (Fig. 5D). All kinds of metal atoms can be captured and stabilized by defective carbon supports simultaneously, thus forming highly dispersed single-atom centers on the supports, named high-entropy single atoms (HESACs) (Fig. 5E). By adjusting the pyrolysis temperature and the amount/types of printing templates, the HESACs with up to 11 metals can be produced (Fig. 5F). It is anticipated that the high-entropy subnanoclusters or single atoms with the highest atomic utilization will be developed using more advanced methods in the future.

### Morphological regulation of HEAs

To further enhance the electrocatalytic activities, a feasible strategy is to design anisotropic morphology, which can not only maximize the number of active sites but also improve the catalytic activity of a single catalytic site through possible ligand/strain effect (*91*). However, conventional high-temperature/shock-cooling techniques make it difficult to maintain the anisotropic structure of nanomaterials. The overwhelming challenge for synthesizing thermodynamically unfavorable anisotropic HEAs is that immiscible mixtures or heterogeneous structures prefer to be formed using conventional synthetic approaches. At present, the dominant way to address this challenge is to introduce appropriate capping agents into mild wet-chemical methods, greatly reducing the formation energy of alloys to promote the growth along specific crystalline orientations (*92*). Several HEAs with special morphologies have been successfully synthesized recently by controlling metals’ growth orientation by adjusting the reaction conditions (*62*, *67*, *93*–*95*). Most of these synthesis techniques highly depend on the preferential reduction of certain metals, which are easy to form targeted structures. Subsequently, the other metals are reduced together on the initial synthetic metallic crystal seeds, and finally, further atomic diffusion enables the formation of HEAs.

**Fig. 6.**
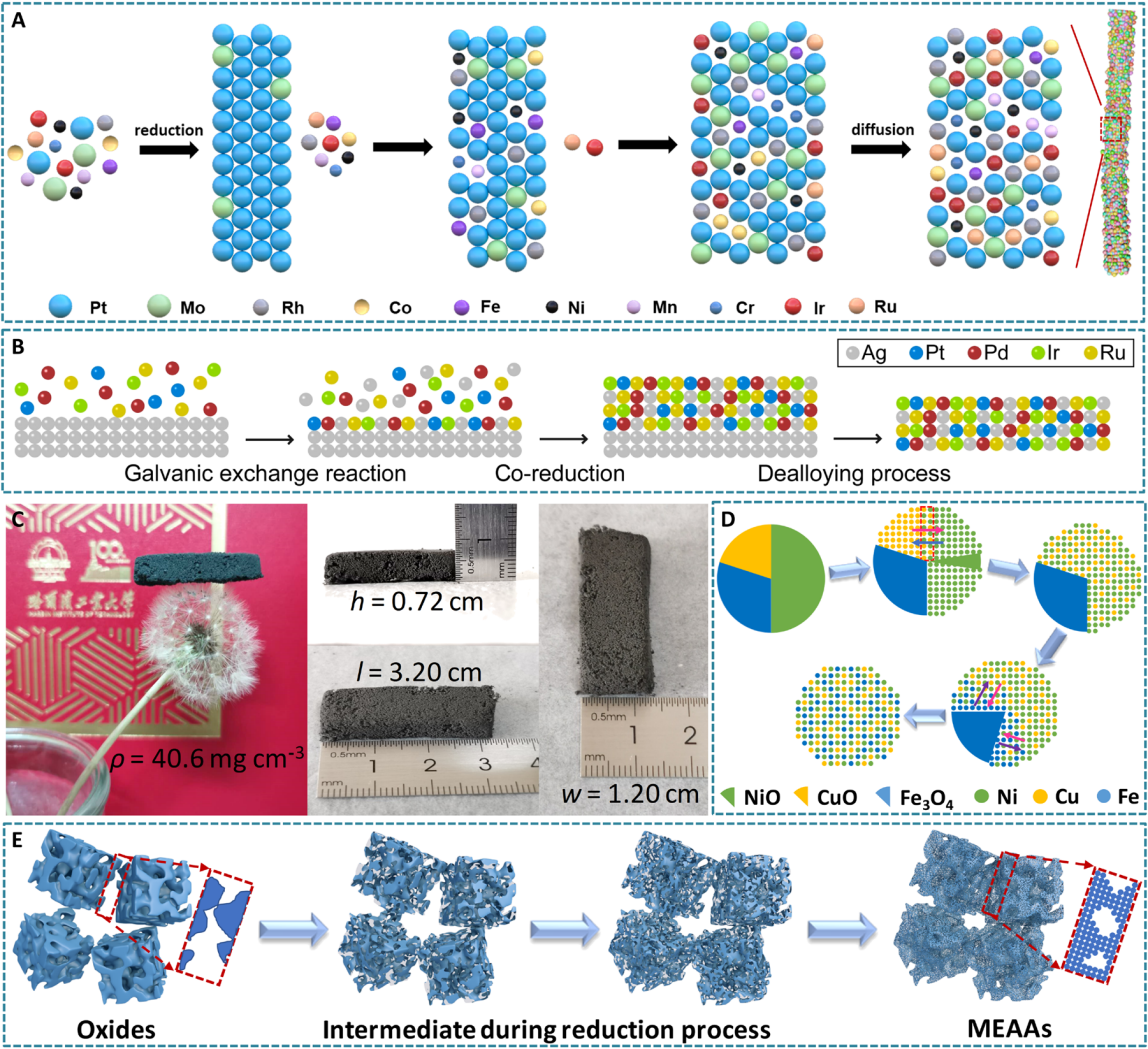
Morphological regulation of HEAs for efficient electrocatalysis. Schematic illustrations for the formation mechanism of (**A**) PtRhMoIrCoRuFeNiMnCr HEA nanowires and (**B**) PtPdIrRuAg HEA subnanometer ribbons. Reproduced with permission from Elsevier (*72*) and the American Chemical Society (*38*). (**C**) Photograph and corresponding sizes of NiCoFeCu MEAAs. Schematic illustrations for (**D**) the formation mechanism and (**E**) morphological evolutions of MEAAs. Reproduced with permission from John Wiley and Sons (*104*).

Generally, CO gases or CO molecules in situ generated from carbonyl-containing compounds (e.g., metal carbonyl compounds, formaldehyde, and formic acid) can be selectively adsorbed on specific crystal facets of metals. This inhibits the growth of certain specific planes of metal nanocrystals, thus facilitating the acquisition of anisotropic nanostructures (*96*). Taking the PtRhMoIrCoRuFeNiMnCr ultrathin nanowires as an example, the sequential reduction mechanism of HEA nanowires was revealed by regulating the feeding of molybdenum hexacarbonyl [Mo(CO)_6_], glucose, dodecyl trimethyl ammonium chloride (DTAC), and oleylamine (Fig. 6A) (*72*). Because of the existence of Mo(CO)_6_, the ultrathin Pt nanowires can be preferentially formed. Then, several alloying elements were reduced one by one according to the different reduction rates of metals as reaction temperatures increased. The continuous reaction ensures the further diffusion and rearrangement of atoms, thus realizing the formation of single-phase solid solution nanowires. Note that several kinds of elements (Cu, Pd, Ag, and Cu) with higher reduction potential cannot participate in the formation of Pt-based HEA nanowires because they have a faster nucleation rate than Pt. By choosing the other elements that reduce slower than Pt, a library of over 200 kinds of HEA nanowires can be constructed by adjusting the categories of metal precursors and reaction temperatures. This work also overthrew the view that single-phase solid solution HEAs can only be formed by co-reduction of all-metal precursors, laying a foundation for developing more ultrathin HEAs. Different from Pt-based alloys, the strong adsorption of CO on the Pd (111) made it easy to form nanosheet morphology. With the further reduction and diffusion of other metal atoms, a series of PdMo-based HEA nanosheets were successfully constructed, thus extending ultrathin nanosheet morphology into HEA systems (*51*, *64*, *93*).

Another important strategy to eliminate the distinction of nucleation/growth kinetics of different metals in HEAs is to use templates to control the anisotropic growth of multimetal nanocrystals. In this respect, several HEA nanowires were synthesized by treating Al-rich nanowires with alkaline solutions to selectively remove most aluminum, such as AlNiCoRuMo and NiPtPdRhIrAl (*97*, *98*). As-synthesized HEA nanowires tend to form porous or hollow structures due to the selective etching of elements with higher electrochemical activity. In comparison, components with lower activity can be diffused and recombined (*99*). In addition, Ag nanowires can serve as templates to construct 2D HEA subnanometer ribbons with a thickness of ~0.8 nm, the thinnest one among HEAs so far (*38*). The time-dependent experiments combined with molecule dynamic simulations revealed the formation mechanism of HEA nanoribbons as follows: (i) the nucleation of different metal atoms by the galvanic exchange reactions, (ii) the co-reduction of different metals, and (iii) the removement of Ag cores via dealloying process (Fig. 6B). Activated Ag and Pd atoms with maximum mean square displacements promote the crystallization and stabilization of HEA shells on Ag templates. This strategy is highly general for ultrathin HENA nanoribbons containing five to eight noble metal elements.

The template method is also an important strategy for constructing 3D porous HEAs (*100*). Two kinds of latex particles, polystyrene (PS) or poly(methyl methacrylate) (PMMA) beads, can serve as templates to realize the preparation of macro- and mesoporous HEAs (*73*). The existence of PS or PMMA templates not only allows metal precursors to be reduced at a relatively low temperature but also freezes the alloy in its high-entropy state by inhibiting the atom diffusion and dealloying during the thermal treatment. These are responsible for the formation of porous HEAs with homogeneous compositions. In addition, a simple dealloying strategy was further presented to fabricate free-standing nanoporous HEAs containing 12 or 16 evenly distributed metal elements (*100*). Specifically, a single solid solution phase Mn-rich precursor alloy was obtained by melting the corresponding pure metals. Then, the excess Mn atoms were removed by acid treatment, resulting in porous HEAs.

The essential issue for forming HEAs is element segregation caused by the reduction rate difference of metal precursors (*101*). However, exerting a certain kind of reaction conditions onto the preparation process is beneficial for driving the reduction potentials of different metals to be in equilibrium (*102*, *103*). This also makes it possible to successfully prepare HEAs. To facilitate the alloying of immiscible metals while maintaining the 3D aerogel structures, the combination of auto-combustion and low-temperature reduction methods were presented to achieve the general preparation of 3D porous medium-entropy alloy aerogels (MEAAs) with ultralow density and ultrahigh porosity (Fig. 6C) (*104*). Metal oxide–based heterostructures were synthesized by the initial auto-combustion procedure, followed by the metal ions being reduced at a low temperature. The ionic level chemical mixing leads to the short-range diffusion of metal atoms, promoting the formation of single-phase alloys (Fig. 6D). Note that the increase in reduction degree makes the skeleton structure more robust, thus forming the aerogel structures (Fig. 6E). Another strategy, the freeze-thaw method, was further proposed to prepare HEA aerogels (HEAAs) with 3D cross-linking network structures and large surface area (*39*). This method can be extended to prepare diverse HEAAs, including but not limited to nine different metallic elements, such as Pd, Cu, Au, Ag, Bi, In, Co, Ni, and Zn.

### Regulating disordering and ordering of HEAs

**Fig. 7.**
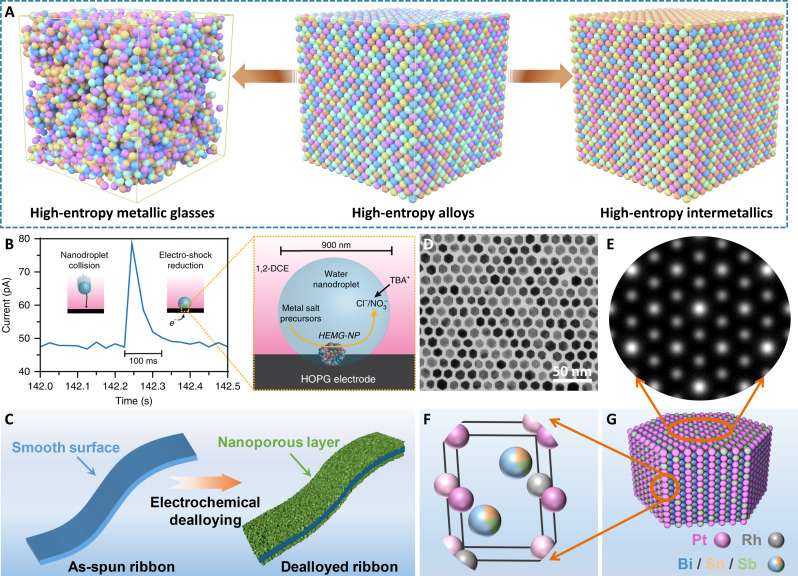
Regulating disordering and ordering of HEAs for efficient electrocatalysis. (**A**) Representative atomic distributions in quinary HEMGs, HEAs, and HEIs. Schematic illustrations for the formation of (**B**) HEA nanoparticles by the nanodroplet-mediated electrodeposition method (current transient corresponding to the collision of a single nanodroplet onto a carbon fiber ultramicroelectrodes (UME) of 4 μm biased at −0.4 V versus Ag/AgCl) and (**C**) nanoporous (FeCoNiB_0.75_)_97_Pt_3_ HEMGs. Reproduced with permission from the Nature Publishing Group (*110*) and John Wiley and Sons (*113*). (**D**) Representative TEM image of PtRhBiSnSb HEI nanoplates. (**E**) Enlarged HAADF-STEM image of the typical Rh-substituted Pt columns. (**F**) Typical structural model of a crystalline unit. (**G**) Schematic illustration showing the atomic arrangement in (PtRh)(BiSnSb) HEI nanoplates. Reproduced with permission from John Wiley and Sons (*37*).

HEAs tend to be single-phase solid solution structures composed of mixed metal components, often showing local order or short-range disorder. However, alloys can also display long-range disordered and long-range ordered structures, usually defined as metallic glasses and intermetallics, respectively (*105*–*107*). Further extended to HEAs, high-entropy metallic glasses (HEMGs) and HEIs have been reasonably developed via engineering the disordering and ordering of atomic arrangement (Fig. 7A) (*108*, *109*). In this respect, the long-range disordered atomic structure, isotropy, and metastability are the key properties of the HEMGs, rendering them with exciting electrocatalytic performance (*105*, *106*). On the contrary, when the atoms of the components occupied the lattice with a specific atomic stoichiometry, they will induce the ordered atomic arrangement in the HEIs to be isotropic or in a specific crystallization direction. Hence, the HEIs always have a long-range atomic ordering (*105*, *107*). Although the ordered structure is an important feature of intermetallics, the site occupancy in each sublattice of HEIs is still random or nearly random due to the more constituent elements than sublattices (*109*). By integrating the advantages of HEAs with metallic glasses or intermetallics, the HEMGs and HEIs have become competitive candidates for the grail war of electrocatalysis. Note that the interconversion between HEMGs and HEIs occurs when the specific conditions are met, such as temperature, stoichiometry, and atom binding environment (*110*–*112*).

Generally, amorphous materials with unique properties can provide disordered atomic arrangement, abundant surface defects and corrosion resistance, as well as dangling bonds, contributing more active sites to catalyze electrochemical reactions. Recently, tremendous research progress has been made in using amorphous features such as high chemical homogeneity and atomic diffusivity to enable the fabrication of HEMGs (*110*, *113*). Electrochemical synthesis can easily control the atomic arrangement, thus becoming an important strategy for stabilizing the thermodynamically nonequilibrium state of amorphous materials. Considering that the input of potential can change the reduction rates of different metals, the nanodroplet-mediated electrodeposition method was developed to prepare HEMGs with disordered and amorphous microstructures (*110*). This process was achieved by the collision of nanodroplets filled with metal chlorides with an ultramicroelectrode caused by a 100-ms electroshock. On the ultramicroelectrodes, a constant potential facilitated this collision, at which time all the metal ions were effectively reduced (Fig. 7B). As-prepared HEAs show amorphous characteristic with highly evenly distributed metal elements. In addition, HEMG ribbon can provide a mechanically strong and flexible substrate to adjust nanoporous and multiphase surface structures (*114*). This stimulates the development of nanoporous HEMGs through a dealloying strategy. For instance, a self-supported (FeCoNiB_0.75_)_97_Pt_3_ HEMG template was first prepared via a scalable and simple melt-spinning method. Then, electrochemical dealloying was used to create the nanoporous surface structures (Fig. 7C) (*113*). The removal of nonfunctional transition metals substantially increases the specific surface area of materials, provides more favorable catalytic active sites, and creates abundant surface defects.

Compared with the traditional solid solution alloy nanocrystals, intermetallic nanocrystals with long-range ordered structures and well-defined compositions can exhibit robust structural properties in electrocatalysis due to the strong atomic interaction (*107*). On the basis of the binary/ternary parent intermetallics, HEIs, whose partial atomic substitution mainly originated from the specific sites of the binary counterparts, have been successfully designed to construct ideal catalytic active sites (*37*, *40*, *115*–*118*). However, the synthesis difficulty from high-temperature annealing to overcoming the energy barrier from disorder to order makes HEIs always a benchwarmer for the grail war of electrocatalysis. An inevitable problem is the bulk and phase-separation structures caused by high-temperature treatment (*116*–*118*). To address this challenge, carbon supports were preadded into metal precursors before annealing at 850°C (*40*). The agglomeration of nanoparticles was prevented due to the tight interaction between nanoparticles and carbon supports. As-prepared (PtIr)(FeCoCu) HEI nanoparticles display a similar crystalline structure to L1_0_-PtFe intermetallics, where partial Pt atoms are replaced by Ir atoms, while Co and Cu atoms occupy the position of Fe atoms. Such harsh preparation conditions further challenge the morphological control of intermetallics. Inspired by the abovementioned morphological regulation of HEAs, the initial formation of Bi-complex nanoplate becomes a favorable template to grow PtBi-based HEIs (*37*). Because of the similar hexagonal close-packed (hcp) structures of PtBi, RhBi, PtSn, and PtSb intermetallics, the diffusion and ordering of Pt/Rh/Bi/Sn/Sb atoms make it possible to form well-defined structurally ordered HEI nanoplates. To this end, (PtRh)(BiSnSb) HEI nanoplates were developed via a typical complexing-reducing-ordering growth mechanism. The preformed Bi-complex nanoplate templates benefit the subsequent reduction and cooperation of Sn and Sb atoms. As a class of fully ordered hcp multimetal nanoplates with the similar crystal structure to hcp PtBi intermetallic, the partial Pt atoms in a crystalline unit are substituted by the Rh atoms, while a portion of Bi atoms are replaced by Sn or Sb atoms (Fig. 7, D to G). The development of these methods provides an effective and feasible strategy for realizing the atomic long-range ordered HEIs, thus promoting the applications of HEIs in energy-related electrocatalysis.

## ELECTROCATALYTIC APPLICATIONS OF HEAS

Since all kinds of high-entropy materials were applied in various heterogeneous catalytic reactions, HEAs have been rapidly developed in electrocatalysis (*31*, *119*–*121*). Because of the several key advantages of HEAs, as discussed in the “Advantages of HEA Electrocatalysts” section, the adsorption energy can be broadened through reasonable electronic hybridization (*25*, *122*, *123*). Therefore, the ligand, strain, or synergistic effect can be optimized to further dominate the electrocatalytic activity, stability, and selectivity of HEAs. By reasonably regulating the surface structures, various HEAs have been applied in the field of electrocatalysis, including water electrolysis electrocatalysis, fuel cell electrocatalysis, and other promising reactions (Table 1). To highlight the importance of HEAs as catalysts in boosting catalytic performance, in this section, several key electrocatalytic reactions were chosen to build the relationship between as-developed HEAs and catalytic activity.

**Table 1.** Electrocatalytic applications of state-of-the-art HEA-based catalysts.

Catalysts	Applications	Activity	Stability	Reference
PtNiFeCoCu nanoparticles	HER in 1.0 M KOH	11 mV @ 10 mA cm^−2^; 10.96 A mg_Pt_^−1^ @ −0.07 V	No attenuation after 10,000 cycles	(*32*)
NiCoFePtRh nanoparticles	HER in 0.5 M H_2_SO_4_	27 mV @ 10 mA cm^−2^; 28.3 A mg^−1^ @ −0.05 V	No attenuation after 10,000 cycles or 100 h @ 10 mA cm^−2^	(*33*)
PdPtRhIrCu metallene	HER in 1.0 M KOH	15 mV @ 10 mA cm^−2^; 1.55 mA cm^−2^ @ −25 mV	No attenuation after 10,000 cycles	(*51*)
PtPdRhRuCu mesoporous nanospheres	HER in 1.0 M KOH	10 mV @ 10 mA cm^−2^; 6.1 A mg_Pt_^−1^ @ −0.05 V	No attenuation after 10,000 cycles	(*63*)
PtPdRhRuCu mesoporous nanospheres	HER in 0.5 M H_2_SO_4_	13 mV @ 10 mA cm^−2^	No attenuation after 10,000 cycles	(*63*)
PtPdRhRuCu mesoporous nanospheres	HER in 1.0 M PBS	28 mV @ 10 mA cm^−2^	No attenuation after 10,000 cycles	(*63*)
PtMoPdRhNi nanocrystals	HER in 1.0 M KOH	9.7 mV @ 10 mA cm^−2^	6.9 mV attenuation in overpotential @ 10 mA cm^−2^ after 5,000 cycles	(*64*)
FeCoNiAlTi intermetallics	HER in 1.0 M KOH	88.2 mV @ 10 mA cm^−2^	No attenuation after 40 h	(*71*)
PdMoGaInNi nanosheets	HER in 0.5 M H_2_SO_4_	13 mV @ 10 mA cm^−2^	No attenuation after 12 h	(*93*)
FeCoNiCuMn nanoparticles	HER in 1.0 M KOH	281 mV@100 mA cm^−2^	No attenuation after 20 h	(*134*)
IrFeCoNiCu nanoparticles	OER in 0.1 M HClO_4_	302 mV @ 10 mA cm^−2^; 34.67 A g_Ir_^−1^@ 300 mV	6.9 mV attenuation in overpotential @ 10 mA cm^−2^ after 12 h	(*74*)
FeCoNiCuMn nanoparticles	OER in 1.0 M KOH	386 mV @ 200 mA cm^−2^	No attenuation after 20 h	(*134*)
PtPdIrRuAg subnanometer ribbons	ORR in 0.1 M KOH	4.28 A mg_Pt+Rh_^−1^; 1.69 A mg_PGM_^−1^	56.5% retention after 30,000 cycles	(*38*)
PtIrFeCoCu intermetallics	ORR in 0.1 M HClO_4_	1.29 A mg^−1^; 4.06 mA cm^−1^	9 mV attenuation in E_1/2_ after 60,000 cycles	(*40*)
L1_2_-FeCoNiCuPd intermetallics	ORR in 0.1 M KOH	2.04 A mg_Pd_^−1^	10 mV attenuation in E_1/2_ after 10,000 cycles	(*66*)
Pt_4_FeCoCuNi intermetallics	ORR in 0.1 M HClO_4_	3.78 A mg_Pt_^−1^	74% retention after 30,000 cycles	(*116*)
PtFeCoNiCuZn intermetallics	ORR in 0.1 M HClO_4_	2.40 A mg_Pt_^−1^; 3.47 mA cm^−2^	94.1% retention after 10,000 cycles	(*118*)
PtPdFeCoNi nanoparticles	ORR in 0.1 M HClO_4_	1.23 A mg_Pt_^−1^; 1.80 mA cm^−2^	6 mV attenuation in E_1/2_ after 50,000 cycles	(*140*)
PtRuNiCoFeMo nanowires	HOR in 0.1 M KOH	6.75 A mg_Pt+Ru_^−1^; 8.96 mA cm^−2^	73.6% retention after 2,000 s	(*36*)
PtRhMoRuIr nanowires	HOR in 0.1 M KOH	5.8 A mg_Pt+Ru_^−1^	81.1% retention after 2,000 s	(*72*)
PdNiRuIrRh nanoparticles	HOR in 0.1 M KOH	3.25 A mg^−1^	81% retention after 3,500 s	(*142*)
PtNiFeCoCu nanoparticles	MOR in 1.0 M KOH +1.0 M methanol	15.04 A mg^−1^	93.6% retention after 1,000 cycles	(*32*)
PtRhBiSnSb nanoplates	MOR in 1.0 M KOH +1.0 M methanol	19.53 A mg_Pt+Rh_^−1^	70.2% retention after 5,000 cycles	(*37*)
PtRhGaNiW nanowires	MOR in 0.5 M H_2_SO_4_ + 2 M CH_3_OH	2.71 A mg_Pt_^−1^; 5.66 mA cm^−2^	72.0% retention after 1,000 cycles	(*148*)
Mo_1_-PdPtNiCuZn nanosheets	MOR in 1.0 M KOH + 1.0 M methanol	24.55 A mg_Pt_^−1^; 11.62 A mg_Pd+Pt_^−1^; 16.55 mA cm^−2^	Excellent stability after 3,600 s	(*149*)
PtRhBiSnSb nanoplates	EOR in 1.0 M KOH + 1.0 M ethanol	15.56 A mg_Pt+Rh_^−1^	~66% retention after 20,000 s	(*37*)
PtRuRhCoNi nanowires	EOR in 1.0 M KOH + 1.0 M ethanol	7.68 A mg_PtRuRh_^−1^; 7.55 mA cm^−2^	Excellent stability after 3,600 s	(*95*)
PtRhBiSnSb nanoplates	GOR in 1.0 M KOH + 1.0 M glycerol	7.54 A mg_Pt+Rh_^−1^	~62% retention after 20,000 s	(*37*)
PtBiPbNiCo nanoplates	FAOR in 0.5 M H_2_SO_4_ + 0.5 M HOOH	7.1 A mg_Pt_^−1^; 27.2 mA cm^−2^	Excellent stability after 1,000 s	(*67*)
PdCuAuAgBiIn aerogels	CO_2_RR 0.5 M KHCO_3_	200 mA cm^−2^ @ −2.0 V_RHE_	No attenuation after 3,600 s at −0.8 V_RHE_	(*39*)
AuAgPtPdCu nanoparticles	CO_2_RR 0.5 M K_2_SO_4_	10.15 mA cm^−2^ @ −0.8 V_RHE_	No attenuation after 1,000 s at −0.9 V_RHE_	(*145*)

### Water electrolyzers

To obtain clean hydrogen energy, water electrolysis technologies are found to be one of the most high-efficient and eco-friendly ones for producing hydrogen fuels under mild conditions and powered by renewable energy (*7*, *8*). However, the current water electrolyzers display a higher cell voltage than that of the theoretical voltage of 1.23 V (*124*). Generally, a desired catalyst with optimized adsorption binding strength for the reaction intermediates, adsorption hydrogen (H*) for HER, and oxygen-containing intermediates (O*, HO*, and HOO*) for OER can generate the most outstanding catalytic activity (*8*, *64*, *87*). The diversity of composition and structure in HEAs allows the continuous regulation of the catalyst surface electronic structures, which is beneficial to breaking the scaling relationships for electrocatalysis, surpassing the long-established not too strong or too weak guidance (*125*–*127*). Therefore, excellent HER performance can be achieved on HEAs, whether they are acidic or alkaline electrolytes.

Since the electrocatalytic activity of HEAs is highly relevant to the selection of constituent elements and proportions, it is necessary to construct the relationship between compositions and electrocatalytic activities. For HER electrocatalysis, a widely accepted idea is to give priority to selecting metals with high activity to pair with other elements according to the required electronic structure. This can effectively adjust the adsorption/desorption behavior toward reaction intermediates, which is closely related to the compositions, thus affecting the catalytic activity. In terms of HER electrocatalysis, Pt and Rh are the key in the binding ability of H* and the H_2_O dissociation, respectively, and transition metals (Fe/Co/Ni) can effectively adjust the electronic structure of active sites. To this end, the PtMoPdRhNi HEA nanocrystals with strong *d-d* electron interaction can be rationally designed to achieve high-efficiency HER electrocatalysis (*64*). By optimizing the compositional distribution, the Pt_28_Mo_6_Pd_28_Rh_27_Ni_15_ nanocrystals displayed the lowest overpotential of 9.7 mV at 10 mA cm^−2^ in 1.0 M KOH electrolyte. In this catalysis, H_2_O dissociation was facilitated by Pt and Ni sites, while the combination of H* to H_2_ was promoted by Pt and Rh sites. Furthermore, the computer-facilitated screening predicted the optimal H* binding energy on PdMoGaInNi, which can be further realized via experimental results where PdMoGaInNi showed the most excellent HER performance among various catalysts with different prerequisites (*93*). These results witnessed the importance of the selection of constituent metals on the catalytic activity of HEAs.

Apart from the optimization of compositions and structures, the complexity of HEAs makes each element display different contributions to electrocatalysis, presenting the site-to-site electron transfer mechanism for the stabilization of intermediates in HEAs (*32*, *49*). This can be reflected in well-dispersed PtNiFeCoCu HEA nanoparticles by showing a high mass activity of 10.96 A mg^−1^_Pt_ at −0.07 V versus reversible hydrogen electrode (RHE) toward HER in 1.0 M KOH electrolyte (*32*). In this catalysis, the obvious downshift of *s* and *p* orbitals in H_2_O molecules reveals that the transfer of active electrons from HEAs to H_2_O induces stable adsorption of H_2_O molecules. The surface Pt atoms are beneficial for electron transfer and act as electron storage for the HER electrocatalysis, while Ni and Co atoms on the surface dominate the electroactive region near the Fermi level. This mechanism can also be observed in the PdFeCoNiCu HEA system (*50*).

In addition, the continuous reduction of particle size is the goal for achieving high-performance electrocatalysis. For this purpose, a series of carbon-supported ultrasmall HEA nanoparticles were developed to demonstrate the excellent HER performance (*33*). Although the ultralow noble metal loading, as-obtained ultrasmall NiCoFePtRh HEA nanoparticles still exhibit a low overpotential of 27 mV at 10 mA cm^−2^ and an ultrahigh mass activity of 28.3 A mg^−1^ at −0.05 V versus RHE. The improved HER performances have also been found in other ultrasmall HEA systems, such as ultrafine nanowires and ultrathin nanosheets. For example, both ultrafine HEA nanowires and ultrathin HEA nanosheets have shown better HER performances than those of corresponding low-entropy counterparts (*72*, *93*).

Theoretical calculations and machine learning have provided an insightful understanding of the electronic structures of HEA electrocatalysts. Although the *d*-band center has rationalized the improved electrocatalysis of unary/binary metal materials, it is not suitable for describing the activity of HEAs with complex structures (*128*, *129*). To close this gap, hard x-ray photoelectron spectroscopy (HAXPES) was used to observe the valence band structure of HEA nanoparticles (*130*). The detected broad and featureless valence band spectrum of IrPdPtRhRu HEA nanoparticles indicates different atomic arrangements with unique local density of states. However, although IrPdPtRhRu nanoparticles have a lower *d*-band center located between Ir and Pt, the turnover frequency (TOF) value is relatively high. This observation is not consistent with the well-accepted correlation between the TOF and the experimental *d*-band center positions. The deviation is mainly derived from the complicated atomic arrangements and the difference in local density of states on HEAs, confirming the potential route for HEAs to break the linear scaling relationship.

**Fig. 8.**
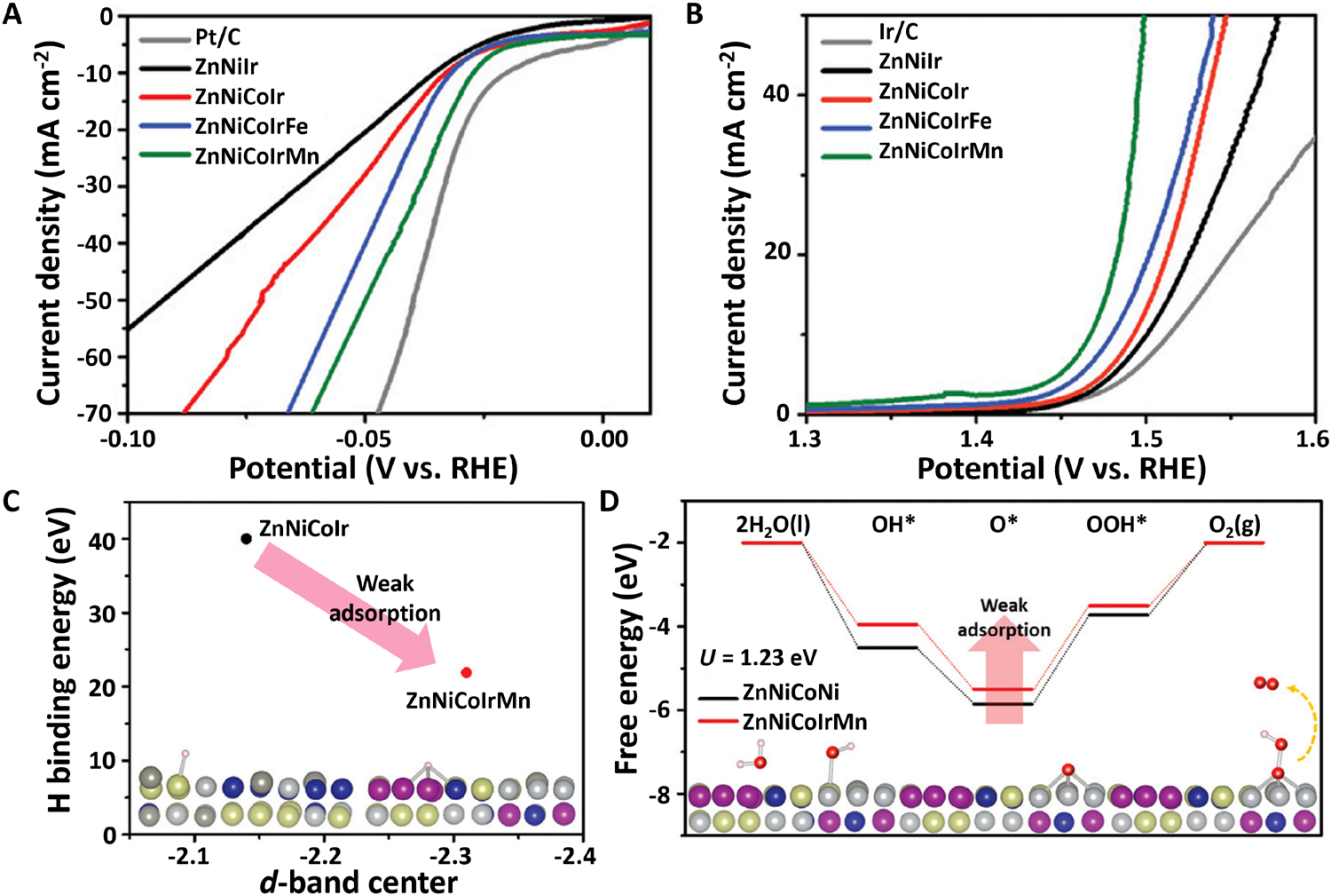
Water electrolyzer–related electrocatalytic applications of HEAs. (**A**) HER and (**B**) OER polarization curves of ZnNiIr, ZnNiCoIr, ZnNiCoIrFe, and ZnNiCoIrMn HEAs and commercial Pt/C or Ir/C catalysts. (**C**) Linear relationship between *d*-band center and hydrogen binding energy on ZnNiCoIr and ZnNiCoIrMn HEAs. (**D**) Energy landscape toward OER intermediates for ZnNiCoIr and ZnNiCoIrMn with *U* = 1.23 V. Reproduced with permission from John Wiley and Sons (*131*).

In terms of OER electrocatalysis, another key half-reaction of water electrolyzers, the most widely accepted catalysts are Ir and Ir-based materials (*7*, *74*). In this case, an important trend is to develop low Ir–containing electrocatalysts with excellent durability and activity for sustainable water electrolyzers. HEAs provide a potential platform for developing low Ir–containing catalysts due to their multicomponent and unique properties. To this end, two kinds of nanoporous Ir-based ZnNiCoIrX (X = Fe, Mn) HEAs with high crystallinity were designed by dealloying with Zn elements as the sacrificial metals (*131*). As-dealloyed ZnNiCoIrMn HEAs decrease hydrogen binding energy (HBE) at the Ir active site relative to LEA and MEA counterparts (Fig. 8A). In addition to accelerating HER dynamics, the ZnNiCoIrMn HEAs also exhibit a low OER overpotential of 237 mV at 10 mA cm^−2^ (Fig. 8B). Moreover, alternating the fifth metallic element X also enables optimizing adsorption/desorption processes toward HER/OER intermediates, mainly caused by the fact that Mn atoms with lower electronegativity can induce more electron-deficient Ir atoms than Fe atoms. Theoretical calculations indicate that the introduction of Mn atoms can not only lower the HBE by a more negative *d*-band center (Fig. 8C) but also weaken the adsorption of OH* and O*, thus leading to a low energy barrier for the formation of HOO* (Fig. 8D).

Because of the completely disordered distribution of metal atoms in HEAs, some metal atoms will lose their own properties, promoting the conversion of metal sites from inactive to active for catalyzing chemical reactions (*132*, *133*). The continuous electronic structure regulation makes the active sites of HEAs more unexpected. To identify the real electrocatalytically active sites of HEAs, low-electronegativity Mn and high-electronegativity Cu atoms were alloyed with Fe, Co, and Ni atoms to form the FeCoNiCuMn HEA nanoparticles (*134*). The difference in electronegativity leads to strong local electron interaction, thus activating inactive Cu atoms into electron-enriched active sites. This can effectively reduce the adsorption energy toward reactants, intermediates, and products and synergistically enhance the bifunctional HER and OER activities. Therefore, the proposed high-entropy atomic environment regulation strategy is anticipated to drive more metals to be suitable for electrocatalytic water splitting, promoting the vigorous development of sustainable water electrolyzers.

The high-entropy and sluggish diffusion effects of HEAs make it tend to form a single solid solution phase, which makes it difficult to generate the potential difference between multiple phases to improve the corrosion resistance (*135*). Therefore, HEA electrocatalysts can display excellent stability during the long-term electrocatalytic tests. To demonstrate the advantages of HEAs as stable catalysts, nonnoble metal-based HEA nanoparticles (FeCoNiCu) were designed to stabilize Pt sites (HEA@Pt) (*136*). In this case, the multielement regulation and entropy stabilization effects made as-prepared HEA@Pt catalysts show the improved stability with negligible attenuation after 100-hour HER stability tests. The enhanced stability can be attributed to the uniform dispersion of strong metal-metal bonds and efficient regulation of Pt′s electronic structure.

### Fuel cells

Polymer electrolyte membrane fuel cells (PEMFCs) have been widely spread in vehicles, portable electronics, smart grids, and military-related fields due to their low operating temperature, high power density, quick start-up, and nonpollution (*5*). ORR is a key cathodic half-reaction of PEMFCs, while its sluggish kinetics severely hinders the further applications of PEMFCs (*4*, *137*). Inspired by the compositional complexity and the heterogeneous surface of HEAs, the electronic structure can be adjusted in a wide range, thus providing a promising platform for alleviating the sluggish kinetics of ORR. The wide binding energy distribution makes HEAs possible to adjust the binding energy of the surface-active sites of HEAs to the optimal value.

A well-accepted criterion for designing highly efficient ORR catalysts is that a desired catalyst should have the optimal bonding strength toward oxygen-containing intermediates (*10*). Although this theory has rationalized ORR activity of single metals or traditional alloys, the known methods of associating adsorption energy are hardly extended to the complex surface of HEAs, including the *d*-band model, the generalized coordination number model, and the orbitalwise coordination number model. To alleviate this issue, a simple model was developed to predict adsorption energies based on the local binding site composition (*138*). By calculating the adsorption energies of *OH and *O on a random subset of IrPdPtRhRu HEA (111) surface sites, the corresponding linear regression models were constructed. The near-continuous adsorption energy of the HEA surface is beneficial for maximizing the ORR activity, which is achieved by tuning the overall compositions of HEAs. In this regard, the optimized Ir_0.102_Pd_0.320_Pt_0.093_Rh_0.196_Ru_0.289_ makes the adsorption energies close to the peak of the volcano curve. In addition, the nudged elastic band model was optimized to reveal the close-knit correlation of the maximum ORR activity and ridges (*139*). By following the ridge of catalytic activity to the edge of the component space, and then replacing one element with another, a desired catalyst with optimal performance can be constructed. There is no doubt that the most catalytically active compositions can be searched along the ridge instead of the whole alloy composition space, thereby narrowing the retrieval range of HEAs for ORR electrocatalysis.

The similar enhancement of ORR performance can also be experimentally observed on various emerging HEAs (*38*, *40*, *54*, *103*, *140*). Considering that the decreased sizes can maximize the surface atomic utilization, sub–3 nm HEAs were tightly anchored on ordered mesoporous carbon by an anchoring-carbonization strategy (*103*). The ultrasmall structure makes it show ultrahigh electrochemically active surface area (ECSA) of 102.3 m^2^ g^−1^, 1.62 times higher than that of commercial Pt/C. In addition to inherent high-entropy and sluggish diffusion effects, as-prepared sub–3 nm HEAs also exhibit stable interface bonding to contribute higher ORR activity compared with that of low- and medium-entropy counterparts. Moreover, due to the synergistic effects of various active sites, HESACs loaded on porous nitride-doped carbon display high ORR activity (*54*). These studies evidence the importance of size effect in HEAs for ORR electrocatalysis, in accordance with that in traditional alloys.

**Fig. 9.**
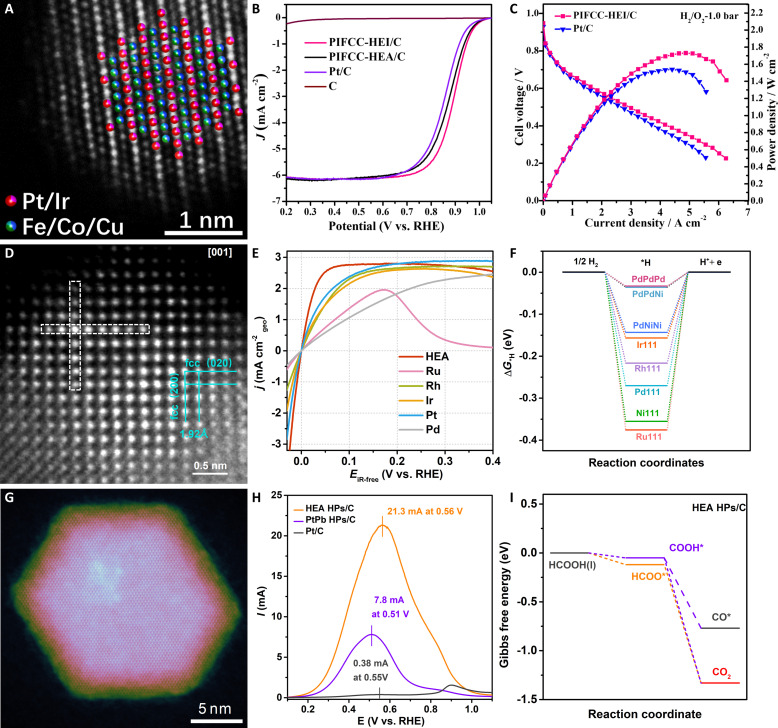
Fuel cell–related electrocatalytic applications of HEAs. (**A**) Atomically resolved HAADF-STEM image of an individual PtIrFeCoNi HEI nanoparticle. (**B**) ORR polarization curves of PtIrFeCoNi HEI and HEA nanoparticles, carbon support, and commercial Pt/C in O_2_-saturated 0.1 M HClO_4_ solution. (**C**) Polarization and power density curves of the H_2_/O_2_ fuel cell with PtIrFeCoNi HEI nanoparticles as the cathode and commercial Pt/C as the anode under 1.0-bar O_2_ pressure. Reproduced with permission from the American Chemical Society (*40*). (**D**) Atomically resolved HAADF-STEM image of PdNiRuIrRh HEA nanoparticle. (**E**) HOR polarization curves of PdNiRuIrRh HEA nanoparticles, the corresponding single-metal counterparts, and commercial Pt/C in H_2_-saturated 0.1 M KOH. (**F**) Adsorption free energies of *H on the hollow-fcc sites of PdNiRuIrRh HEA nanoparticles and pure metal (111) facets. Reproduced with permission from John Wiley and Sons (*142*). (**G**) HAADF-STEM image of an individual PtBiPbNiCo core/shell HEA nanoplate. (**H**) FAOR curves of PtBiPbNiCo core/shell HEA nanoplates, PtPb nanoplates, and commercial Pt/C in 0.5 M H_2_SO_4_ + 0.5 M HCOOH. (**I**) Gibbs free energy diagram of PtBiPbNiCo core/shell HEA nanoplates toward FAOR electrocatalysis. Reproduced with permission from John Wiley and Sons (*67*).

The morphological regulation also affords the possibility to optimize the binding strength of catalysts toward the reaction intermediates, necessitating combining the HEAs with reasonable morphological control. Taking 2D HEAs as an example, the enhanced ORR activity has been reflected on quinary PtPdIrRuAg HEA nanoribbons (*38*). In alkaline electrolytes, the PtPdIrRuAg HEA nanoribbons show 21.0 times higher mass activity than that of commercial Pt/C. In this catalysis, the strong orbital couplings can be achieved on PtPdIrRuAg, and the broadest coverage of Ru-4*d* and Ir-5*d* orbitals increases the electron density near *E*_F_ within PtPdIrRuAg. As a result, the high activity is mainly attributed to the existence of elements with strong reduction ability (Pt, Pd, and Ag) and the promotion of electron transfer between different sites by relatively inert oxidizing elements (Ir and Ru). In addition, in light of the superior structural stability of atomically ordered intermetallics, PtIrFeCoNi HEI nanoparticles were further demonstrated to serve as efficient ORR catalysts, especially in the practical H_2_/O_2_ fuel cells (Fig. 9A) (*40*). The ORR activity of well-designed HEI nanoparticles was not only superior to that of commercial Pt/C but also more excellent than that of disordered counterparts (Fig. 9B). An ultrahigh peak power density of 1.73 W cm^−2^ can be achieved on the practical fuel cells with HEI catalysts as cathodic catalysts (Fig. 9C). The construction of HEI catalysts endowed (001) facets with ultrahigh activity, substantially decreasing the ORR activation barriers and optimizing the *d*-band center.

Another key half-reaction in PEMFCs is hydrogen oxidation reaction (HOR), which mainly occurs within a fast kinetics in acidic conditions (*141*). However, the HOR in an alkaline environment is much more sluggish than that in acidic media, even two orders of magnitude lower (*138*). In this case, the tailorable active sites of multiple elements adjacent to each other allow HEAs to generate more unique properties in HOR electrocatalysis (*36*, *72*, *142*). To clarify the mechanism of the complex atomic structure of HEAs on the HOR electrocatalysis, PdNiRuIrRh HEA nanoparticles were prepared, where the five metal atoms were uniformly dispersed to form a single-phase solid solution (Fig. 9D) (*142*). The PdNiRuIrRh HEA nanoparticles exhibit a high HOR mass activity of 3.25 A mg^−1^ in alkaline electrolytes, much higher than that of single-metal counterparts (Fig. 9E). In this system, Pd-Pd-Ni/Pd-Pd-Pd bonds dominate the PdNiRuIrRh HEA nanoparticle surface. Therefore, the formation of Pd-Pd-Ni/Pd-Pd-Pd bonds and the presence of Ni/Ru oxophilic sites can optimize the adsorption/desorption toward *H and *OH intermediates, enhancing HOR activity and stability (Fig. 9F).

In addition, ultrathin structures make the alloyed nanowires expose more surface-active sites, thus enhancing electrocatalytic performance. Among numerous ultrathin HEA nanowires, PtRuNiCoFeMo shows the highest HOR mass activity of 6.75 A mg_Pt+Ru_^−1^ in 0.1 M KOH solutions, 2.8, 4.1, and 19.8 times higher than that of HEA nanoparticles, commercial PtRu/C, and Pt/C, respectively (*36*). In addition, the HEA nanowires also exhibit high anti-CO poisoning ability during HOR processes, as well as enhanced stability. Experimental and theoretical analysis revealed that the strong interactions between various metal sites in PtRuNiCoFeMo HEA nanowires can optimize the binding energy of *H and/or *OH intermediates, thereby accelerating the alkaline HOR kinetics. Note that the HOR catalytic activity of such HEA nanowires is also higher than that of low- and medium-entropy alloy nanowires (*72*). It is anticipated that the entropy engineering will make great achievements in HOR electrocatalysis.

Compared with hydrogen energy, liquid fuels can be conveniently handled, stored, and transferred (*143*). It is therefore presented to replace hydrogen fuels with high–energy density liquid fuels to drive the anodic oxidation reactions (*143*). Coupling these electrooxidation reactions with the cathodic ORR aforementioned, direct alcohol/formic acid fuel cells (DAFCs/DFAFCs) can be assembled to convert chemical energy into electrical energy. However, it is difficult to achieve complete oxidation of polyalcohols/formic acid into CO_2_ under mild conditions. Alloying is an important strategy to tune the electronic structure to enhance AOR/formic acid oxidation reaction (FAOR) electrocatalysis, leaving a broad research space in the elemental selection, interaction, and determination of active sites. HEAs have been demonstrated to be a promising candidate for the complete oxidation of these small molecules with favorable electronic and coordination environments.

Various alcohols can be oxidized through a mechanism of the alcohol molecule adsorption and the further oxidation of CO. However, the sluggish kinetics of traditional metal catalysts limits the further applications of alcohol oxidation reactions. In addition, beyond methanol, the cleavage of the C─C bonds and further complete oxidation of the alcohols to CO_2_ are serious problems. HEAs can integrate the respective advantages of multiple elements, further accelerating reaction kinetics by multisite synergistic mechanisms. In this regard, PtRhBiSnSb HEI nanoplates not only show higher activity toward methanol oxidation reaction (MOR) compared with that of low-/medium-entropy counterparts but also exhibit greater advantages in polyalcohol oxidation reactions, such as ethanol oxidation reaction (EOR) and glycerol oxidation reaction (GOR) (*37*). The improved electron transfer by the additional Rh atoms and the synergistic protection of Bi, Sn, and Sb sites are two key factors for the excellent AOR performance of PtRhBiSnSb HEI nanoplates.

In terms of stability, the adsorption of intermediates (CO_ads_) makes the catalysts to be poisoned due to the occupation of catalytic active sites by CO_ads_. To address this issue, a variety of oxophilic metals have been used to accelerate the electrooxidation of CO-containing intermediates, such as Rh, Cu, Ga, and Ru (*85*, *143*). On the basis of traditional binary/ternary alloy catalysts, HEAs can further alleviate the poisoning through the diluted metal-metal bonds and multifunctional coordination environment. Within PtNiFeCoCu HEA nanoparticles, Pt atoms served as the electron reservoir for electrocatalysis, while Ni and Co atoms provided the electron depletion center and the other metal atoms alleviated the energy barrier of electron transfer (*32*). In addition, the well linear correlation of *s* and *p* orbitals of dominant intermediates guaranteed the proton and electron transfer in MOR electrocatalysis. The energy barrier of rate-determining step was lowered due to the site-to-site electron transfer, and CO poisoning was eliminated. Therefore, the mass activity of as-synthesized PtNiFeCoCu nanoparticles only decayed by 6.4% after 1000 MOR cycles, more stable than that of commercial Pt/C.

Compared with the other fuels, the low toxicity, storability, high boiling point, and transportability of formic acid drive the widespread spread of DFAFCs. The PtBiPbNiCo HEA hexagonal nanoplates with a unique structure of medium-entropy core/high-entropy atomic layer shells show excellent FAOR electrocatalytic performance (Fig. 9G) (*67*). Note that the mass activity of PtBiPbNiCo HEA core/shell nanoplates was as high as 7.1 A mg_Pt_^−1^ in 0.5 M H_2_SO_4_ containing 0.5 M formic acid, much larger than that of traditional binary nanoplates and commercial Pt/C (Fig. 9H). The DFAFCs using such nanoplates and commercial Pt/C as anodic and cathodic catalysts respectively display the highest power density of 321.2 mW cm^−2^, as well as outstanding stability. The reaction path for FAOR of core/shell nanoplates was a direct formate pathway instead of the indirect pathway, demonstrating the favorable dehydrogenation reaction of HEA core/shell nanoplates. CO_2_ can be produced by the sequential dehydrogenation of HEA core/shell nanoplates, inhibiting CO poisoning. The high FAOR activity of HEA core/shell nanoplates mainly originated from the inhibition of dehydration way and the optimization of two dehydrogenation procedures (Fig. 9I). Note that the increased entropy and lattice distortion effects of HEAs are important for enhanced electrocatalysis.

### Carbon dioxide reduction reactions

Converting greenhouse CO_2_ into value-added chemical fuels electrochemically is another important technology for carbon-neutral economy (*9*). A wide range of C_1_ products (including CO, HCOOH, HCHO, CH_3_OH, and CH_4_) and C_2_ products (including C_2_H_4_, CH_3_CHO, C_2_H_5_OH, and CH_3_COOH), as well as various C_2+_ (including n-C_3_H_7_OH) can be selectively obtained from the conversion of CO_2_ based on the electrochemical processes (*9*, *144*). However, this process is kinetically sluggish due to the complicated reaction mechanism, thus necessitating highly active and selective HEA CO_2_RR electrocatalysts with adjustable configuration space (*144*, *145*).

Cu and Cu-based materials have been considered as feasible catalysts to convert CO_2_ into value-added C_2_/C_3_ products, yet the pristine Cu catalysts tend to exhibit low activity and selectivity due to the competition of HERs (*9*). Combining density functional theory calculations with supervised machine learning has predicted that two Cu-based HEAs, CoCuGaNiZn and AgAuCuPdPt, can serve as promising candidates for CO_2_RR (*144*). The CO and H adsorption energies on the (111) surfaces of CoCuGaNiZn and AgAuCuPdPt HEAs were calculated, and the optimized sites with weak hydrogen adsorption and strong CO adsorption can facilitate the high selectivity of HEAs. Furthermore, machine learning models were developed to forecast the adsorption energies of several important CO_2_RR intermediates (COOH*, CO*, and CHO) by considering 1280 adsorption sites on the designed FeCoNiCuMo HEAs (*146*). Because of the difference in adsorption features toward COOH*, CO*, and CHO* on HEAs, the scaling relation between the adsorption energy of COOH* (CHO*) and CO* has been circumvented. Therefore, the designed HEA active sites can achieve low potential toward CO_2_RR. These results show that the adsorption energy of key intermediates can be regulated by reasonable component design, thereby optimizing the selectivity and activity of HEAs for CO_2_RR. Experimentally, AuAgPtPdCu HEA nanoparticles with fcc structures were demonstrated as an efficient CO_2_RR model catalyst (*145*). The adsorption energy of two key intermediates (*OCH_3_ and *O) on the surface of Cu (111) and HEAs was reversed, inducing a stronger thermodynamic advantage of HEAs compared with pristine Cu metals. Therefore, the AuAgPtPdCu HEA nanoparticles show a high Faraday efficiency of 100% at 0.3 V (versus RHE) toward gaseous hydrocarbons. It has been proved theoretically and experimentally that HEAs can effectively promote the electrocatalytic CO_2_RR process by optimizing the adsorption energy of the key reaction intermediates of CO_2_RR.

Pd-based electrocatalysts have shown high selectivity toward HCOOH and CO during CO_2_RR electrocatalysis, while the promotion of CO tolerance and the inhibition of HER are still very urgent (*147*). In this regard, senary PdCuAuAgBiIn HEAAs can achieve almost 100% Faraday efficiency of C_1_ products from −0.7 to −1.1 V (versus RHE) and a highest value of 98.1% toward HCOOH at −1.1 V versus RHE (*39*). This is mainly because of the regulation of electronic structures by the strong interactions between different metals and the surface unsaturated sites. The optimized adsorption of *HCOO intermediates guarantees the enhanced electrocatalysis, making the activity of HEAAs not only more excellent than that of nanoparticle counterparts but also outperforms Pd metallic aerogels. Note that the multicomponent advantages of HEAs make it possible to diversify the products of CO_2_RR electrocatalysis, which is expected to obtain unique activity and selectivity different from conventional alloys.

## SUMMARY AND OUTLOOK

HEAs, an emerging branch of alloying materials, have shown tremendous potential to overcome the species limitations determined by the differences in intrinsic physical and chemical properties between different metal atoms. The continuous adjustment of surface electronic structure is the most typical characteristic of HEAs, which potentially enables the break of scaling relationship in electrocatalysis. In this review, we summarize the latest progress of HEA electrocatalysts with two important trends of diminishment and multidimensionality over the past few years. We demonstrate the advantages of HEA electrocatalysts from the aspects of high entropy, nanometer, and multidimension. In addition, advanced characterizations have been used to disclose the structural complexities of HEA electrocatalysts, especially establishing the synergetic mechanism between the multisites in HEAs and electrocatalytic properties. We also emphasize the structural regulation strategies for maximizing electrocatalytic performance, including size, morphology, and atomic arrangement engineering. Despite the attractive material platforms for various electrocatalytic reactions, the development of HEAs in electrocatalysis is still in its infancy, and enormous challenges need to be tackled in the future. Several key challenges are as follows:

1) Controlled preparation and tailoring of HEAs. Although the morphology-controlled synthesis of HEAs has been realized and reported, the controllable morphologies of HEAs are very limited. The electronic structure regulation strategies for traditional alloys, such as doping engineering, defect engineering, and surface and interface engineering, have yet been extended to HEAs. Especially, the differences in reduction potentials and miscibility behavior of different metals further add difficulties to the fine structural regulations. Therefore, developing more controllable synthetic approach is a major task in this field and is potentially breaking through the activity limitation of HEAs.

2) The extension to other electrocatalytic applications. Considering the advantageous multisite synergistic catalysis of HEAs, it provides additional opportunities for steering electrocatalytic reactions with high degree of complexity and specifically improving the selectivity. For example, the increase in element identity makes the change of products more diversified for organic electrosynthesis and the electrocatalytic conversion of organic molecules, thus potentially enabling the exclusive synthesis of high-value-added products. As these reactions often require multiple adsorption sites for multiple elementary steps, HEAs could serve as the ideal model catalysts.

3) Understanding the relationship between structure and catalytic activity in HEAs. The complexity of the surface and internal structure of HEAs, in addition to the surface dynamics during electrocatalysis, severely challenges the identification of active sites of HEA-based electrocatalysts. To address this challenge, it is necessary to leverage advanced characterizations for the analysis of atomic structure and in situ spectroscopy for tracking the surface adsorbates during electrocatalysis. The ensemble effect of highly diverse surface sites should also be carefully considered, because each surface site has its sensitivity to elementary reaction steps. This calls for a precise establishment of atomic models in experimental and theoretical investigation to unravel and quantitate ensemble effect.

4) Integrating HEAs into industry. The structural merits drive the rapid development of HEAs in electrocatalysis but remain too early for practical applications. Moving toward industry, the following aspects of HEAs need to be addressed: scalability, economic feasibility, reproducibility, controllability, and sustainability.
